# Sex‐specific floral attraction traits in a sequentially hermaphroditic species

**DOI:** 10.1002/ece3.5987

**Published:** 2020-02-05

**Authors:** Kristen Peach, Jasen W. Liu, Kristen N. Klitgaard, Susan J. Mazer

**Affiliations:** ^1^ Department of Ecology, Evolution and Marine Biology University of California, Santa Barbara Santa Barbara CA USA

**Keywords:** bisexual flowers, dichogamy, floral color, floral evolution, hermaphrodite, pollination, sexual dimorphism

## Abstract

● Many angiosperms are hermaphroditic and produce bisexual flowers in which male (pollen export) and female (stigma receptivity) functions are separated temporally. This sequential hermaphroditism may be associated with variation in flower size, color, or pattern, all of which may influence pollinator attraction. In this study, we describe variation in these traits across discrete functional sex stages within and between 225 greenhouse‐grown individuals of *Clarkia unguiculata* (Onagraceae). In addition, to identify the effects of floral phenotype on pollinator attraction in this species, we examine the effects of these floral traits on pollen receipt in ~180 individuals in an experimental field array.
● Petal area, ultraviolet (UV)‐absorbing nectar guide area, and blue and green mean petal reflectance differ significantly across the functional sex stages of *C. unguiculata*. Male‐ and female‐phase flowers display significantly different pollinator attraction traits. Petal and UV nectar guide area increase as flowers progress from male phase to female phase, while blue reflectance and green reflectance peak during anther maturation.
● In field arrays of *C. unguiculata*, female‐phase flowers with large UV nectar guides receive more pollen than those with small nectar guides, and female‐phase flowers with high mean blue reflectance values are more likely to receive pollen than those with low blue reflectance. Female‐phase flowers with green mean reflectance values that differ most from background foliage also receive more pollen than those that are more similar to foliage. These findings indicate that components of flower color and pattern influence pollen receipt, independent of other plant attributes that may covary with floral traits. We discuss these results in the context of hypotheses that have been proposed to explain sex‐specific floral attraction traits, and we suggest future research that could improve our understanding of sexual dimorphism in sequentially hermaphroditic species and the evolution of features that promote outcrossing.

## Graphical abstract

This study is one of the first to apply predictions of sexual dimorphism (previously applied to dioecious or trioecious species) to the functional sex stages of a bisexual flower. We suggest future research that could improve our understanding of sexual dimorphism in sequentially hermaphroditic species and the evolution of features that promote outcrossing.

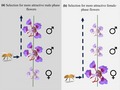

## 1 INTRODUCTION

Sexual reproduction occurs via a wide variety of reproductive modes, including unisexuality and hermaphroditism (sequential, serial, and simultaneous) (Desjardins & Fernald, 2009; Heule, Salzburger, & Böhne, 2014). Most flowering plant species produce bisexual flowers (~90%), with pollen‐producing (male) and ovule‐producing (female) structures contained in the same flower (Barrett & Hough, 2013). In many of these species, pollen export and stigma receptivity are separated by time (dichogamy) (Barrett & Hough, 2013), such that, at any given moment, individual flowers are functionally unisexual. Of the remaining species, ~4% are monoecious, producing unisexual flowers of both sexes on the same individual, and ~6% are dioecious, in which individual plants are exclusively either male or female (Barrett & Hough, 2013; Renner & Ricklefs, 1995). The evolution of dioecy is commonly associated with morphologically distinct male and female flowers (Charlesworth, 1999; Darwin, 1877). Such sexual dimorphism is thought to evolve in response to sex‐specific differences in the strength or direction of selection on individual traits or in the cost of reproduction (Barrett & Hough, 2013; Delph & Ashman, 2006).

In plants, sexual dimorphism has been defined as the condition in which primary and secondary sexual characters differ between male and female individuals (Barrett & Hough, 2013; Dawson & Geber, 1999). Primary sex characters are those that are essential for reproduction; in plants, these are the androecium (stamens) and gynoecium (carpels) (Geber, 1999). By contrast, secondary sex characters are those that affect the likelihood of mating but are not essential for reproduction; these include floral attraction traits that promote pollinator visitation in outcrossing taxa (Geber, 1999). This definition of sexual dimorphism has restricted its examination (in plants) mostly to dioecious or trioecious species. Among zoologists, however, the definition of sexual dimorphism has been more expansive. For example, investigators of sequentially hermaphroditic fish species describe the male and female phases of individuals as being sexually dimorphic (Ijiri et al., 2008; Yoshinaga et al., 2004). In some of these species, a single fish retains both male and female reproductive organs throughout its life, although only one type of organ is functional at a given time. For example, in *Paralichthys olivaceus*, semen and eggs may be obtained from either mature males or females, and both testes and ovaries are present in all mature individuals (Fan et al., 2014; Sun et al., 2009). Accordingly, sexual dimorphism may comprise morphological differences (e.g., sex‐specific body size or coloration) between the male and female phases of a single individual across its lifetime (Desjardins & Fernald, 2009). Similarly, species that produce sequentially hermaphroditic flowers may exhibit differences in secondary sex traits between the male and female phases of their bisexual flowers (Davis et al., 2014; Jabbari, Davis, & Carter, 2013). Differences in floral traits (secondary sex traits) between male‐ and female‐phase flowers may (a) be a significant source of intraspecific phenotypic variation in floral traits and/or (b) result in one sex‐specific phenotype being more attractive to pollinators than the other (Davis et al., 2014).

**Figure 1:**
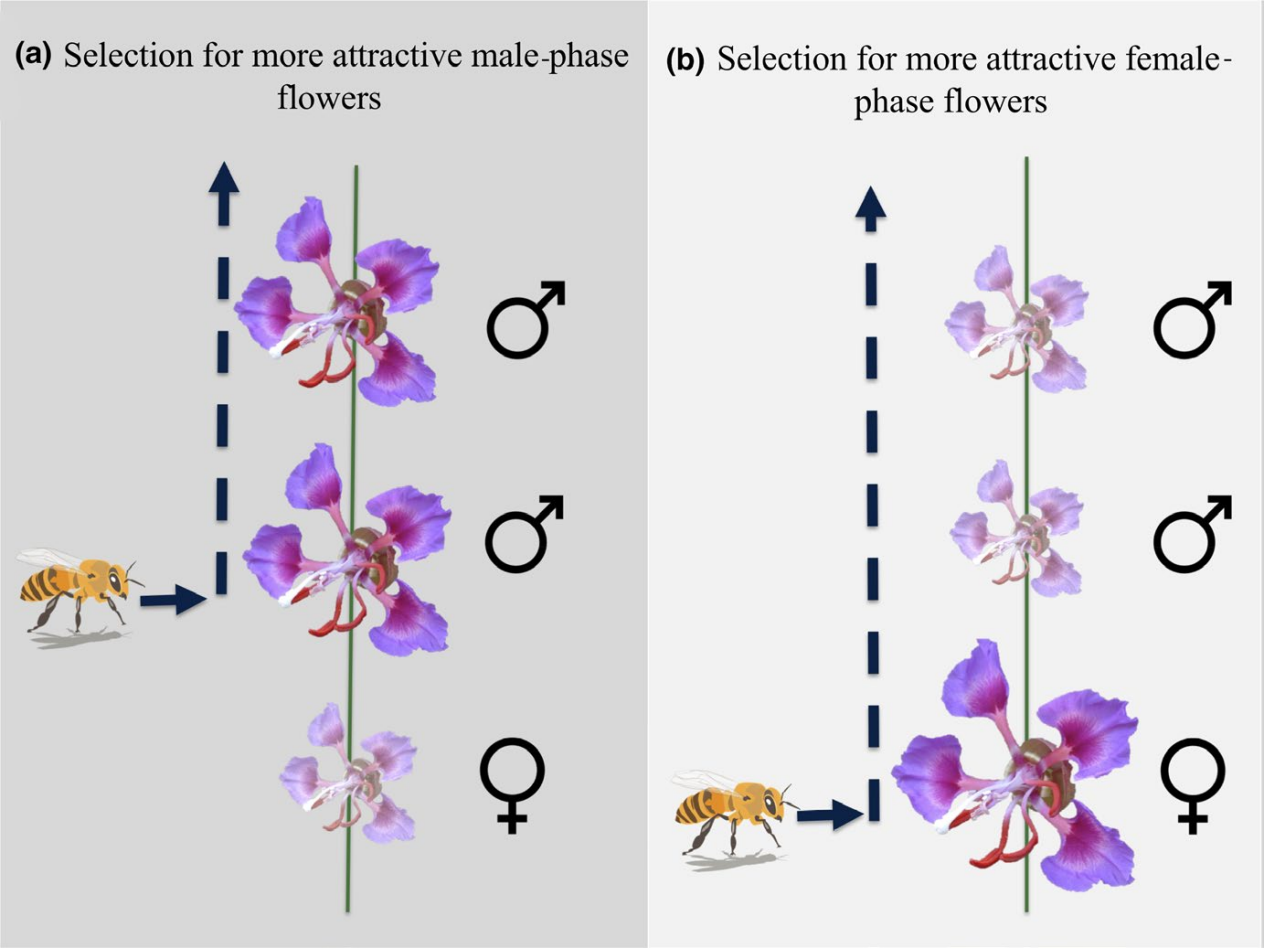
Graphic visualization of how two fitness functions may influence the evolution of sexual dimorphism in *Clarkia unguiculata* (where flower size is used as a proxy for floral attractiveness). (a). Male‐phase flowers may require more visits from pollinators to successfully export all of their pollen than female‐phase flowers need to receive adequate pollen for all available ovules. (b). More attractive female‐phase flowers could discourage self‐pollination by directing pollinator foraging behavior from female‐phase flowers to male‐phase flowers

Sequential sex expression allows for the evolution of different morphologies that may maximize fitness in two (nonmutually exclusive) ways: (a) Male‐phase flowers may require more visits from pollinators to export all available pollen than female‐phase flowers need to fertilize all available ovules, resulting in selection for more attractive male‐phase flowers (especially if excessive pollinator visitation results in negative consequences for female fitness such as pollen clogging or stigma damage) (Harder, Barrett, & Cole, 2000; Lloyd & Webb, 1986), or (b) in species with bisexual flowers and acropetal floral development, more attractive female‐phase flowers may direct pollinator foraging behavior in a way that reduces within‐individual self‐pollination. If the costs associated with self‐fertilization are high, then we may expect selection for more attractive female‐phase flowers (see detailed explanation below and Figure 1).

These two hypotheses for how sexual dimorphism may manifest in a sequentially hermaphroditic plant species are associated with opposing predictions. The first suggests that male‐phase flowers will be more attractive to pollinators than female‐phase flowers, and the second (minimization of geitonogamy) suggests the opposite. By describing sexual dimorphism between the sex stages of a bisexual flower, and identifying floral traits that are attractive to pollinators, we can determine which sex stage is more attractive to pollinators (and therefore which of these pathways has more empirical support).

Changes in flower size or coloration as flowers mature may occur as a result of ontogenetic processes unrelated to functional sex (Krizek & Anderson, 2013). During the “expansion” phase of organ growth, petal cells may increase in size due to increases in the size of the vacuole, resulting in an increase in petal size and/or dilution of pigments within the vacuole (Krizek & Anderson, 2013; Powell & Lenhard, 2012). If selection is neutral for flower size and color, then significant differences between the functional sex stages of bisexual flowers would be simply the byproduct of floral development. However, flower petals are usually considered “fully developed” at the time of reproductive maturity, so changes in flower size after the onset of anther maturity should be considered distinct from initial organ growth. Moreover, the energetic cost to color change suggests a functional value to this trait (Weiss & Lamont, 1997).

### 1.1 Selection for more attractive male‐phase flowers

In primarily outcrossing species, male or male‐phase flowers may have evolved to be more attractive than female or female‐phase flowers because competition for pollinators should favor attractive displays more strongly in the former (Ashman, 2000; Delph & Ashman, 2006; Yakimowski, Glaettli, & Barrett, 2011). Several studies suggest that attractive traits tend to benefit male function more than female function because more pollinator visits are typically required to export all available pollen than to receive enough pollen to fertilize all available ovules (Bell & Hamilton, 1985; Stanton, Snow, & Handel, 1986; Young & Stanton, 1990). Some studies have even shown that particularly high rates of pollinator visitation may incur a cost to female fitness in the form of damaged carpels and a reduction in seed set (Aizen et al., 2014; Sáez, Morales, Ramos, & Aizen, 2014). These sex‐specific visitation requirements may contribute to the evolution of sexual dimorphism in bisexual flowers in the form of more attractive male‐phase flowers (Figure 1).

### 1.2 Selection for more attractive female‐phase flowers

Self‐fertilization is not entirely prevented by dichogamy, as pollen grains can be transferred from flower to flower within an individual plant (geitonogamy). Flower‐ and plant‐level traits may reduce the likelihood of geitonogamous self‐fertilization in a synergistic way (floral positioning and sexual dimorphism).

Species with temporal separation of sex roles in which male function precedes female function (i.e., protandrous) are most commonly acropetalous (flowers closest to the base of the stem develop first, Figure 1), resulting in female‐phase flowers occurring below male‐phase flowers on the stem (the opposite arrangement is very rare) (McKone, Ostertag, Rauscher, Heiser, & Russell, 1995). This floral positioning may reduce geitonogamous self‐fertilization if bee pollinators typically forage upward from the base of the stem to the apex, thereby encountering female‐phase flowers prior to male‐phase flowers, which they often do (Harder et al., 2000) (Figure 1). Given this sequence of floral visitation, pollinators are less likely to transfer pollen between two flowers on the same plant relative to a case in which they visit male‐phase flowers prior to female‐phase flowers (Best & Bierzychudek, 1982; Harder et al., 2000).

Sex‐specific floral phenotypes (i.e., sexual dimorphism) may act synergistically with floral position to minimize geitonogamy. If female‐phase flowers are more attractive to pollinators (e.g., larger or more distinct from background foliage) than male‐phase flowers, then pollinators may preferentially visit female‐phase flowers first. Harder et al. (2000) showed that when pollinators visit male‐phase flowers first, geitonogamous self‐fertilization increases, causing a significant reduction in seed set. To minimize self‐fertilization while still attracting pollinating insects, hermaphroditic plant species may have evolved to produce female‐phase flowers that are more attractive to their primary pollinators than the same flowers during male phase (Figure 1).

To explore the causes and consequences of sex‐specific variation in floral traits, we designed this study with three major goals: (a) to evaluate whether functional sex (stage) is a source of variation in petal size, color, and pattern in *Clarkia unguiculata* (Onagraceae); (b) to determine whether a suite of floral traits that have been assumed to attract insect visitors affects pollen receipt; and (c) to synthesize this information in order to understand the selection regime that may have contributed to sexual dimorphism in this species.

## 2 MATERIALS AND METHODS

### 2.1 Which floral traits are attractive to bees?

A first step toward describing the relative “attractiveness” of flowers in different functional sex stages (or from different individuals or populations) is to quantify flower color and pattern as they are perceived by pollinating insects. Observations from 43 species of Hymenoptera reveal maximal receptor sensitivity for hymenopteran vision at 340 nm (UV), 430 nm (blue), and 535 nm (green) (Dyer et al., 2012; Dyer, Garcia, Shrestha, & Lunau, 2015; Papiorek, Rohde, & Lunau, 2013; Skorupski & Chittka, 2010).

Several studies have shown that the spatial patterns in which flowers reflect and absorb UV light specifically affect the behavior of potential pollinators. A variety of floral UV‐absorption patterns (e.g., “nectar guides” or “bullseyes”) may attract and guide insects, promoting efficient pollination (Horth, Campbell, & Bray, 2014; Leonard & Papaj, 2011). In addition, the size of the UV floral guide has been shown to positively influence pollinator visitation rate and pollen transfer efficiency (Horth et al., 2014; Leonard & Papaj, 2011).

Variation in petal spot pattern can also alter pollinator visitation rates and reproductive success (Eckhart, Rushing, Hart, & Hansen, 2006; Jones, 1996a; 1996b). One study of C*larkia xantiana* ssp. *xantiana* (Onagraceae), which is polymorphic for the presence of petal spots, found frequency‐dependent pollinator preferences for spotted versus unspotted morphs (Eckhart et al., 2006). While individuals of *C. xantiana* are readily identified as spotted versus unspotted, the petal spots of *C. unguiculata* (when present) vary in size and color and shape, which makes it difficult to sort them into discrete categories.

Many studies of variation among individuals in flower color examine and report discontinuous variation (Jones, 1996b; Shipunov, Kosenko, & Volkova, 2011; Whibley et al., 2006) by, for example, using human vision to bin phenotypes into color or pattern classes such as “spotted” versus “unspotted” or “white” versus “purple.” Continuous variation in flower color caused by subtle differences in the composition and concentration of floral pigments is also measured (Papiorek et al., 2013; Tastard, Andalo, Giurfa, Burrus, & Thébaud, 2008). An alternative to reporting a discrete color polymorphism is to use spectrophotometry (Arista, Talavera, Berjano, & Ortiz, 2013; Schemske & Bierzychudek, 2001) or a combination of ocular assessment and spectrophotometry (Berardi, Hildreth, Helm, Winkel, & Smith, 2016; Casper & La Pine, 1984). However, photographic techniques and image analysis can also be useful tools for recording complex floral phenotypes (Brito, Weynans, Sazima, & Lunau, 2015; Del Valle, Gallardo‐López, Buide, Whittall, & Narbona, 2018; Verhoeven, Ren, & Lunau, 2018).

Quantifying variation in continuous floral color and pattern in a way that captures multiple properties of pigment (e.g., UV nectar guide area and reflectance) may improve our ability to identify visual cues used by pollinators in decision‐making, and, when combined with field studies designed to detect their effects on plant performance, help to explain the ecological function of flower color variation or sexual dimorphism within wild plant species. To capture and to quantify this kind of complex variation, we have used a combination of modified photographic methods and a customized ImageJ plug‐in to describe floral color, pattern, and size in a native California forb.

Specific reflectance values of petal color may serve a dual function. First, they may be inherently attractive to pollinators; second, they may make it easier for pollinators to identify flowers against a vegetative background. In the current study, flowers that receive the most pollen in our field array may have color phenotypes that make them readily identifiable as *Clarkia* flowers (and aid their “*Clarkia* bee” specialist pollinators in locating them) or more apparent against an oak woodland background. To begin to understand which of these functions may be driving the patterns observed in this study, we also quantified color‐specific reflectance of background vegetation at our field site to determine whether “attractive” floral color phenotypes are context‐dependent.

### 2.2 Multispectral photography and image analysis

**Figure 2:**
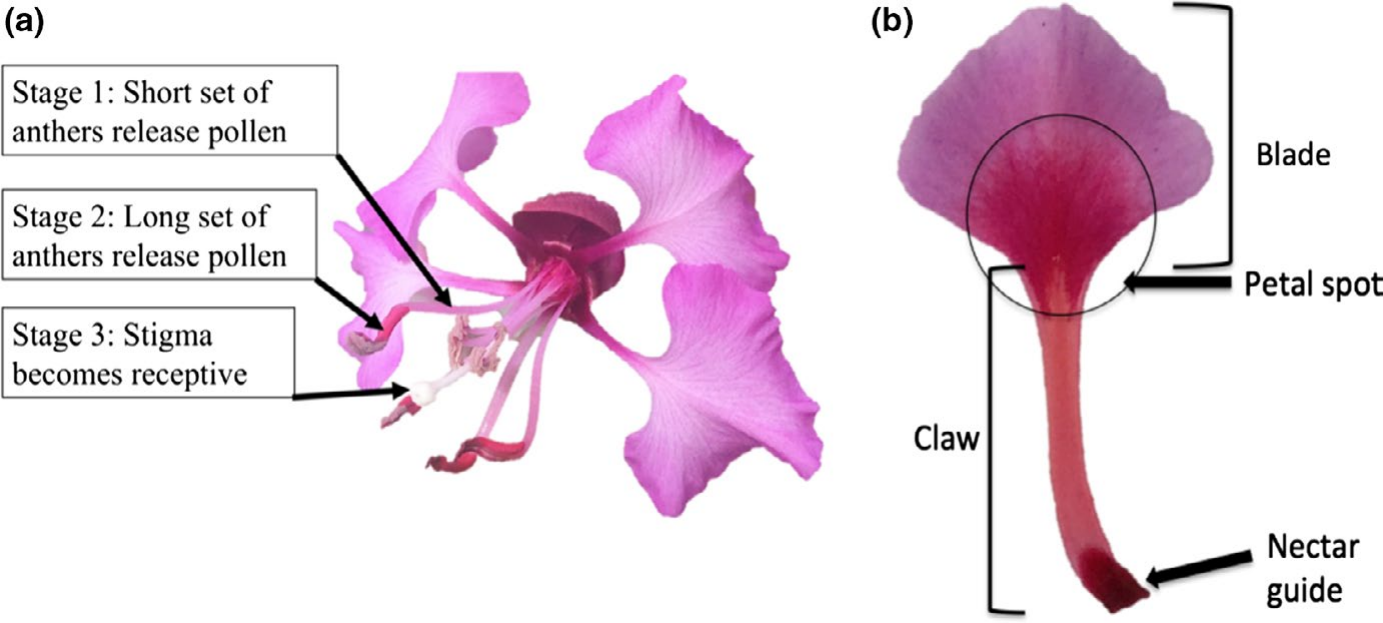
(a) *Clarkia unguiculata* has two whorls of stamens with 4 stamens per whorl. We describe 3 distinct functional sex stages as follows: (1) the anthers of the inner set of stamens mature first, (2) the second set of anthers mature several days later, and (3) the stigma becomes receptive 5–7 days after the outer set of anthers has split open, releasing its pollen. (b). Regions of interest from which objective measurements of blue, green, and ultraviolet reflectance were made

To capture and to quantify complex variation in color‐specific light absorption and reflection, we customized a digital camera (Panasonic LUMIX GX7, http://www.panasonic.com, Kadoma, Osaka Prefecture, Japan) and software plug‐in originally created for the characterization of complex eggshell patterns (Troscianko & Stevens, 2015). The Multispectral Image Analysis Toolbox plug‐in (Troscianko & Stevens, 2015) for ImageJ (Schneider, Rasband, & Eliceiri, 2012) combines photographs taken in visible and ultraviolet wavelengths into one multispectral image and facilitates the extraction of objective measurements of color‐specific reflectance values and pattern information. Four distinct regions of interest (or ROI: petal, claw, blade, and UV nectar guide, Figure 2) were delineated by using ROI tools in ImageJ. UV nectar guide area was determined by viewing the multispectral image in the UV channel and drawing a line around the border of the visually apparent UV‐absorbing nectar guide in the claw of the petal. Objective measurements of mean blue (430‐500 nm), green (510‐530 nm), and ultraviolet (300‐400 nm) reflectance were reported for each region of interest for each petal analyzed (*n* = 225). Area (mm^2^) was reported for each region.

Petal spots of *C. unguiculata* may be either white or purple. The petal spot size was measured as the size of the largest marking (of any color) in the blade region (see Figure 2) and assigned to a “bin” between 1 and 14 (14 being the largest size class, 1 being the smallest) (Appendix 3) in ImageJ. Image acquisition methods are described in detail in Appendix 3.

### 2.3 Study species

*Clarkia* is a genus of self‐compatible, annual herbs native to the western United States (Lewis & Lewis, 1955). The focal species of this study, *C. unguiculata* (Onagraceae), is relatively widespread in California and has been the focus of studies of geographic variation (Jonas & Geber, 1999), pollen performance (Mazer et al., 2018; Németh & Smith‐Huerta, 2003), and mating system (Ivey, Dudley, Hove, Emms, & Mazer, 2016).

*C. unguiculata* occupies oak woodland, grazed or disturbed hillsides, and road cuts in the Coastal Ranges and Sierra Nevada foothills, ranging south to the Peninsular Ranges. Outcrossing rates in field populations in the southern Sierra Nevada range from 0.64 to 0.98 (Ivey et al., 2016). *C. unguiculata* relies on a variety of Hymenopteran pollinators for successful seed production but mostly the solitary “*Clarkia*” bees (Lewis & Lewis, 1955; MacSwain, Raven, & Thorp, 1973).

### 2.4 Three distinct functional sex stages

We classified the developmental stages of the flowers of *C. unguiculata* into three distinct phases (male stage 1, male stage 2, and female stage 3). In stage 1, the inner whorl of anthers matures and releases pollen. Several days later, the outer whorl of anthers matures and releases pollen (stage 2). Five to seven days after stage 2, the stigma becomes receptive (stage 3). We divided the male phase into two distinct stages because in *C. unguiculata* the inner whorl of anthers (stage 1) produces pollen with significantly higher performance than the outer whorl (Peach & Mazer, 2019). Heterantherous species such as *C. unguiculata* have been proposed to exhibit a “division of labor” whereby “feeding anthers” (which produce pollen that may be consumed by an insect) are distinguished from “reproductive anthers” (which produce pollen more likely to contribute to reproduction). In some heterantherous species, feeding anthers can be distinguished from reproductive anthers by the poor performance of their pollen (Mori, Orchard, & Prance, 1980; Nepi, Guarnieri, & Pacini, 2003). Because the two whorls of anthers in *C. unguiculata* may have evolved to serve distinct functions, we treated the dehiscence of each whorl of anthers as a distinct male stage.

### 2.5 Greenhouse study

Seeds from eight wild populations of *C. unguiculata* were collected and cultivated for this study (see Appendix 1 for locations). In 2015–2016, we sampled seeds from 35 maternal families per population. Seeds were placed in coin envelopes (one maternal family per envelope), which were stored in plastic zip‐lock bags with silica desiccant in a dark refrigerator (at 5°C) until use. In fall of 2016, ten seeds per maternal family (35 maternal families x 8 populations) were germinated in agar in 5‐cm Petri dishes. Petri dishes holding dormant seeds were placed in a dark refrigerator for one week to promote germination. After germination, one seedling per maternal family was planted in a cone‐tainer (20.32 cm length, 3.81 cm width SC10 cone‐tainers, http://www.stueweandsons.com, Tangent, OR, USA) filled with a customized soil mixture (5:1:1:1 Sunshine Grow #5, sand, worm castings, http://www.islandseed.com, Santa Barbara, CA, USA). Cone‐tainers were placed in racks in a greenhouse and bottom‐watered for the duration of the study. Plants were grown under controlled temperature (10–15.5°C nighttime temperature range and a 12.7–29.4°C daytime temperature range) and light conditions. Racks of plants were grown under Lumigrow Pro LED lights (http://lumigrow.com Emeryville, CA) for 10 hr a day for the duration of the study. Trays of plants were rotated every week to avoid any potential effects of greenhouse position on focal floral traits.

**Figure 3:**
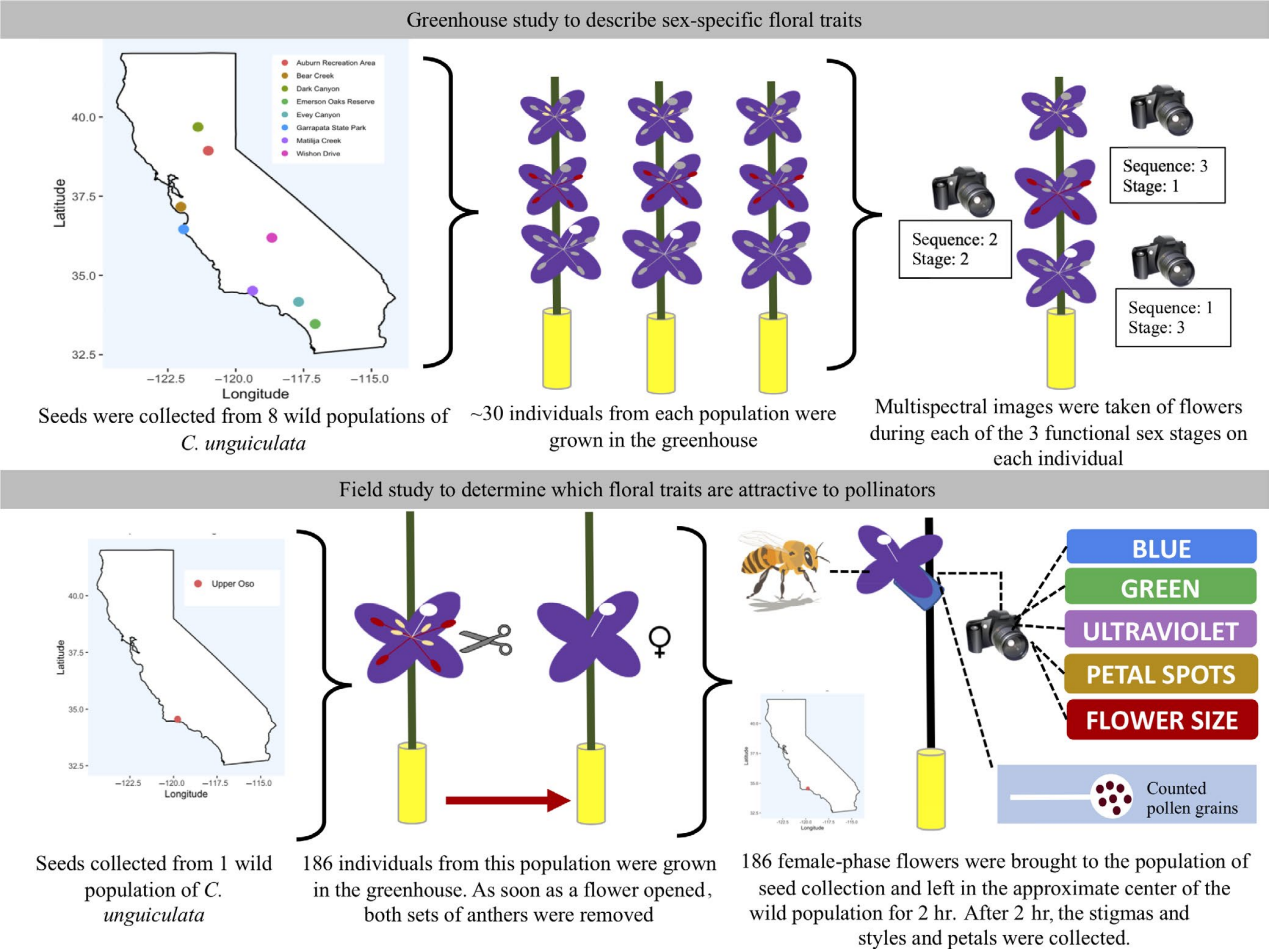
Schematic illustrating the experimental design of the greenhouse and field studies used

Individual petals of three flowers per individual were photographed using a custom multispectral digital camera (see section below and Appendix 3). Each of the three functional sex stages shown in Figure 2 is represented by one petal per individual (1 petal from 3 different flowers). We also recorded the floral sequence of each flower from which petals were sampled. The floral sequence refers to the position of the focal flower relative to the first flower produced on the primary stem (e.g., the third flower produced on the primary stem has a floral sequence of three). We only included data from individual plants from which we had data for each floral sex stage. See Figure 3 for a detailed schematic of the experimental design for the greenhouse and field study.

### 2.6 Field study

In 2016, seeds from individual plants were collected from a field population near Upper Oso Campground in Los Padres National Forest (California, USA; 34.540196, −119.798978) and stored in coin envelopes (one maternal family per envelope). In May of 2018, two siblings per maternal family (*n* = 100 families) were germinated, planted, and grown in the greenhouse in the manner described above. All plants were grown under Lumigrow Pro LED lights (http://lumigrow.com Emeryville, CA, USA) for 10 hr a day. To prevent self‐fertilization, all flowers produced by this cohort were emasculated (anthers removed with scalpel and forceps before dehiscence) the day that the flower opened.

On four days between 13 June 13 and 22 June 2018, a total of 186 flowers were placed in experimental arrays in Los Padres National Forest near Upper Oso Campground. On each day, ~45 “pollen recipient” flowers (each with a fully receptive stigma free of pollen grains) were removed from the greenhouse‐raised plants and placed in a small water pick (7.62‐cm water tubes, http://www.oasisfloralproducts.com, Kent, OH, USA). Flowers were severed at the base of the ovary to provide enough plant tissue to anchor the plant securely in the water pick. To ensure that each recipient flower did not encounter pollen between the greenhouse and the field site, recipient flowers were covered with a perforated segment of a hollow, partially transparent, plastic straw (10mm diam., http://www.bobadirect.com, Arlington Heights, IL, USA) during transportation to the field site. The perforations in each straw were large enough to allow air to enter, but excluded insect visitors; the top and bottom openings of the straw were loosely packed with cotton balls to exclude insects. Upon arrival at the field site, the straw segments were removed and nectar was removed from all flowers using a microcapillary tube (20‐µl calibrated microcapillary tube, http://www.sigmaaldrich.com) and aspirator. The water tubes were then taped to a stiff wire stake (simulating a stem) at an angle similar to that of a naturally borne flower (1 tube per stake). These metal “stems” were arranged in racks and placed in the approximate center of a flowering field population for two hours (between 12:00 p.m. and 2:00 p.m. PST). Each “stem” was assigned a number associated with its position in the field. This “array position” number was included in the final analyses to determine whether a flower's physical position in the field had an effect on pollen receipt. After two hours, the stigma and style from each pollen‐receiving flower were removed and placed in a microcentrifuge tube with formalin acetic acid to arrest pollen tube growth. Each sample was rinsed in DI water three times and then soaked in diluted NaOH for three hours to soften. The stigma was then severed from the style at its base using a razor blade, placed on a microscope slide, and stained with Alexander's stain (Kearns & Inouye, 1993).

Given that some of the pollen deposited on the stigma may have been dislodged when placed in solution in the microcentrifuge tube after they were harvested, the number of pollen grains adhering to the surface provides a measure of the number of grains that became anchored to the stigma within two hours of pollination (the number that began to germinate). A dissecting microscope was used to visualize and count the number of pollen grains deposited on each stigma. One petal from each recipient flower was removed and photographed (using the methods described above and in Appendix 3) immediately upon removal from the field. We also took a multispectral photograph of the background vegetation directly surrounding the field array on each day and extracted mean reflectance values of the background foliage (to compare with petal color reflectance values as described below).

### 2.7 Statistical analyses

#### 2.7.1 Flower color: Independent effects of floral stage, population, and floral sequence

We conducted three‐way fixed effect ANOVAs to detect the independent effects of floral stage (shown in Figure 2), population (from which seeds were collected), floral sequence (of the sampled flower, see Figure 3), and their 2‐way interactions on the mean reflectance of blue, green, and UV light in the petal. Significance testing for each main effect was conducted using type III sum of squares. Only significant interactions were included in the models reported in Table 1. All other interactions were nonsignificant, did not improve model fit, and were therefore excluded from all models presented here. Tukey's HSD (honestly significant difference) tests were conducted to determine whether least square means were significantly different from each other.

**Table 1:** Summary of multivariate models to detect the independent effects of floral sex stage, population and floral sequence on petal area, proportion nectar guide, nectar guide area (mm^2^), and blue, green, and UV mean petal reflectance

Source	Petal area (mm^2^)				Blue reflectance of the petal				Green reflectance of the petal			
	*df*	Sum of Squares	F Ratio	Prob > *F*	*df*	Sum of Squares	F Ratio	Prob > *F*	*df*	Sum of Squares	F Ratio	Prob > *F*
Floral Stage	2	2,446,793	218.98	<.0001*	2	4,760	6.83	.001*	2	4,033	6.55	.002*
Population	7	1,048,677	26.82	<.0001*	7	6,599	2.7	.011*	7	6,042	2.8	.008*
Floral Sequence	1	646	0.12	.73	1	2,292	6.58	.011*	1	2,847	9.25	.003*
Floral Sequence x Population	7	193,425	4.95	<.0001*	7	5,800	2.38	.023*	7	6,975	3.24	.003*
Model	17	3,947,903	41.57	<.0001*	17	23,464	3.96	<.0001*	17	22,949	4.38	<.0001*
Error	207	1,156,471			207	72,170			207	63,737		
Total	224	5,104,375			224	95,634			224	86,686		
			Adj. *R* ^2^: 0.75				Adj. *R* ^2^: 0.18				Adj. *R* ^2^: 0.20	

Source	Proportion nectar guide				Nectar guide area (mm^2^)				Ultraviolet reflectance of the petal			
	*df*	Sum of Squares	F Ratio	Prob > *F*	*df*	Sum of Squares	F Ratio	Prob > *F*	*df*	Sum of Squares	F Ratio	Prob > *F*
Floral Stage	2	0.02	2.17	.12	2	30,323	6.99	.001*	2	324	3.45	.034*
Population	7	0.19	6.1	<.0001*	7	86,892	5.73	<.0001*	7	1924	5.84	<.0001*
Floral Sequence	1	0.02	5.23	.02*	1	2,418	1.12	.29	1	2	0.06	.806
Floral Stage x Population									14	1,301	1.98	.021*
Model	10	0.28	6.4	<.0001*	10	133,227	6.15	<.0001*	24	3,388	3	<.0001*
Error	214	0.93			214	463,715			200	9,407		
Total	224	1.21			224	596,942			224	12,795		
			Adj. *R* ^2^: 0.20				Adj. *R* ^2^: 0.19				Adj. *R* ^2^: 0.18	

* Indicates significant *P* values

#### 2.7.2 Petal area and floral pattern: Independent effects of floral stage, population, and floral sequence

We conducted three‐way fixed effects ANOVAs (using type III sum of squares) to detect the independent effects of floral stage, population, floral sequence and their 2‐way interactions on petal area, UV nectar guide area, and the proportion of the petal occupied by the nectar guide (proportion nectar guide). Only significant interactions were included in the models reported in Table 1. Petal spot size was determined by sorting petal spots into discrete size categories, therefore; we could not treat it was a continuous variable (Appendix 4). Petal spot size was not normally distributed. We conducted an ordinal logistic regression to determine the independent effects of floral stage, floral sequence, and population on petal spot size (Appendix 4).

#### 2.7.3 Effects of flower size, pigment, and pattern on pollen receipt

Among the flowers used in this field study (*n* = 186), pollen receipt ranged from 0 to 285 pollen grains (mean = 35, *SD* ± 54, CV = 169, SE ± 4.06). Pollen receipt (the # of pollen grains counted on each stigma) was non‐normally distributed. We log‐transformed pollen receipt using the log(x + 1) function to preserve meaningful “0” count data (flowers that received 0 pollen grains). To determine the independent effects of blue, green, and UV mean petal reflectance, petal area, and proportion nectar guide on pollen receipt, we conducted a fixed effect ANOVA (using type III sum of squares). Date (of field observations) and array position were initially also included in the model as fixed effects (using the “ANOVA” function from the R package “lmerTest”). Only significant variables were included in the final model, and results are reported in Table 2. To confirm that there was no effect of petal area on pollen receipt, we also conducted a linear regression that included only petal area as the independent variable. We found that petal area had a nonsignificant effect on pollen receipt (*p* = .76).

**Table 2:** (a) Summary of multivariate models to detect the independent effects of proportion nectar guide, and blue and green mean petal reflectance on pollen receipt (treated as a continuous variable) (b) Summary of multivariate models to detect the independent effects of blue and green mean petal reflectance and the absolute value of their difference from the background habitat on pollen receipt (treated as a continuous variable).(c) Summary of generalized linear mixed model fit by maximum likelihood (Laplace approximation) to determine the independent effects of blue, and green mean petal reflectance, and proportion nectar guide area on pollen receipt (>0 pollen grains received)

(2a) Pollen receipt					(2b) Pollen receipt				
Source	*df*	Sum of Squares	F Ratio	Prob > *F*	Source	*df*	Sum of Squares	F Ratio	Prob > *F*
Green mean petal reflectance	1	35.79	13.75	.0003*	Difference in green reflectance between petal and background	1	42.40	15.41	.0001*
Blue mean petal reflectance	1	32.43	12.46	.0005*	Difference in blue reflectance between petal and background	1	37.74	13.72	.0003*
Nectar guide area (mm^2^)	1	24.47	9.4	.0025*					
Model	3	70.96	9.09	<.0001*	Model	2	42.7	7.76	.0006*
Error	172	447.70			Error	173	475.96		
Total	175	518.65			Total	175	518.65		
			Adj. *R* ^2^: 0.12					Adj. *R* ^2^: 0.07	

(2c) Pollen receipt				
Fixed Effects	Estimate	SE	Z value	Pr (>|z|)
Green mean reflectance (Petal)	−2.828	0.9325	−3.033	0.00242*
Blue mean reflectance (Petal)	2.7061	0.929	2.913	0.00358*
Nectar guide area (mm^2^)	0.6517	0.2647	2.462	0.01383*
				Pseudo‐R^2^: 0.21

* Indicates significant *P* values

#### 2.7.4 Generalized linear mixed model

Log‐transforming non‐normal data to meet the normality assumption of linear regression and analysis of variance is common practice in ecology (Bolker, 2008; Schielzeth, 2010). However, some studies suggest that generalized linear mixed models (GLMMs) provide a more appropriate alternative for analyzing non‐normal data when the dependent variable is count data (Quinn & Keough, 2002; Schielzeth, 2010). To ensure that the results of our ANOVA (Table 2a) were accurate, we conducted a generalized linear mixed model fit by maximum likelihood (Laplace approximation) to determine the effects of blue, green, and UV mean petal reflectance, petal area, UV nectar guide area size, and proportion nectar guide on pollen receipt. We considered any pollen receipt over 0 pollen grains to represent a pollinator visit. Individuals that received 0 pollen grains were scored as 0 (failure), and all individuals that received ≥1 pollen grain were scored as 1 (success). We refer to this independent variable as the “probability of pollen receipt” (Table 2b). The mixed effects model was fit using the “glmer” function from the R package “lme4,” and using the binomial family (Bates, Maechler, Bolker, & Walker, 2015).

Flower color (blue, green, and UV reflectance), petal area, nectar guide area, and proportion nectar guide were treated as fixed effects, while standardized array position was treated as a random effect. As trait values often differed from each other by several orders of magnitude, we used the “scale” function to center each predictor at 0 and to obtain Z‐score values of observations for use in model fitting. Nectar guide area, proportion nectar guide, and blue and green mean petal reflectance were the only variables to significantly influence pollen receipt, so all other variables were removed from the final model. Proportion nectar guide was not included in the final model to avoid redundancy with nectar guide area. When proportion nectar guide was included in the model instead of nectar guide area, the direction of its effect on pollen receipt was the same (*p* < .02). The default Wald P‐values are reported here (Table 2).

#### 2.7.5 Difference between petal color and background vegetation

To determine whether the difference in color‐specific reflectance between petals and the background influenced pollen receipt, we conducted a three‐way fixed effects ANOVA (type III SS) to detect the independent effects of the difference in green and blue mean reflectance between the background vegetation and the petal, and date (of the field study) on pollen receipt (treated as a log‐transformed, continuous variable) (Table 2).

## 3 RESULTS

As flowers shifted from male to female phase, all focal traits changed significantly, with the exception of petal spot size and proportion nectar guide (Table 1). In addition, all floral traits differed significantly among populations, and several were influenced by floral sequence and by the interaction between these variables or by the interaction between floral stage and population (Table 1). Below, we describe the phenotypic changes that occur as flowers mature, and the effects of floral attraction traits on pollen receipt.

### 3.1 Effects of stage on floral attraction traits

**Figure 4:**
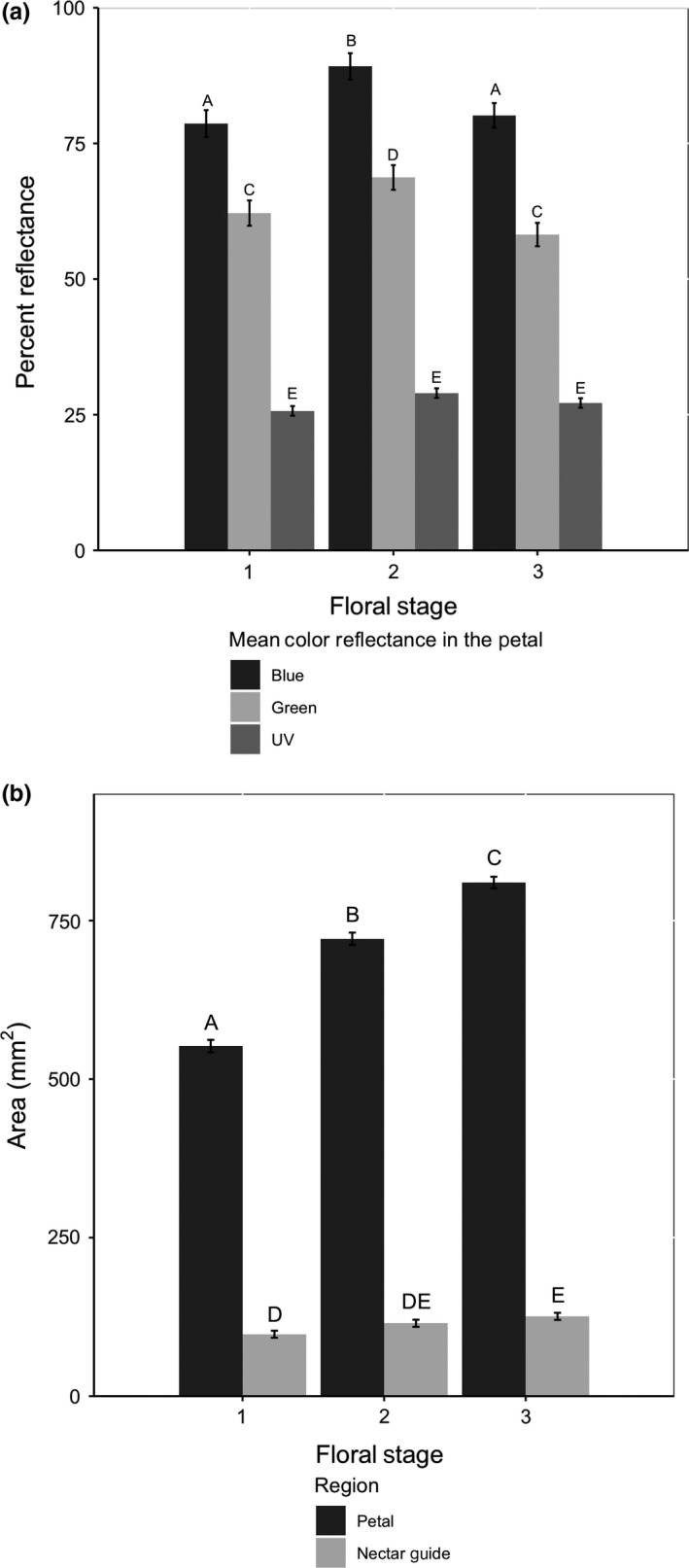
Different letters indicate significant differences between floral sex stages in one of the focal floral attraction traits (differences between least square means). Bars indicate standard error. (a) Floral sex stage had a significant effect on blue, green, and UV mean petal reflectance independent of all other factors in the model. (b) Floral sex stage had a significant effect on petal area (mm^2^) and UV nectar guide area (mm^2^) of petals of *Clarkia unguiculata*

Petals and UV nectar guides were largest in stage 3 (female) flowers. Mean blue, green, and UV reflectance showed nonlinear transitions across functional sex, with peak reflectance values occurring in stage 2 (male) (Figure 4). Floral stages differed significantly with respect to blue, green, and UV mean petal reflectance, independent of the other factors tested (Table 1). Petals sampled during stage 1 had lower blue (least square mean = 78.65 ± 2.47 SE), green (62.15 ± 2.32 SE), and UV (25.70 ± 0.89 SE) mean petal reflectance than petals sampled during stage 2. Petals sampled during stage 2 had significantly higher blue (89.19 ± 2.42 SE) and green (68.71 ± 2.27 SE) mean reflectance than petals sampled during stage 3 (blue: 80.14 ± 2.29 SE, green: 58.19 ± 2.15 SE).

Floral stage had a significant effect on both petal area and UV nectar guide area (Figure 4), independent of variation in the other factors in the model. Mean petal area increased significantly between each pair of successive stages (stage 1 mean petal area = 552.03mm^2^ ± 9.90 SE; stage 2 mean petal area = 721.35mm^2^ ± 9.68 SE; stage 3 mean petal area = 810.03mm^2^ ± 9.17 SE) (Figure 4). UV‐absorbing nectar guide area also increased as flowers matured; nectar guides of stage 1 flowers were significantly smaller (97.62 ± 5.59 SE) than those of stage 3 flowers (125.82 ± 5.63 SE). The parameter estimates for models summarized in Table 1 are reported in Appendix 2. The independent effects of population and floral sequence on focal floral attraction traits are reported in Table 1 and described in Appendix 4.

### 3.2 The effects of petal area, nectar guide area, and B/G/UV reflectance on pollen receipt

Flowers with low levels of green reflectance or high levels of blue reflectance receive more pollen than those with high green or low blue reflectance (Table 2). Nectar guide area had a significant positive effect on pollen receipt (*p* = .02). Proportion nectar guide (when included in the model instead of nectar guide area) also has a significant positive effect on pollen receipt (*p* = .02). We did not detect a significant independent effect of UV mean reflectance, petal spot size, or petal area on pollen receipt in either the ANOVA or GLMM.

**Figure 5:**
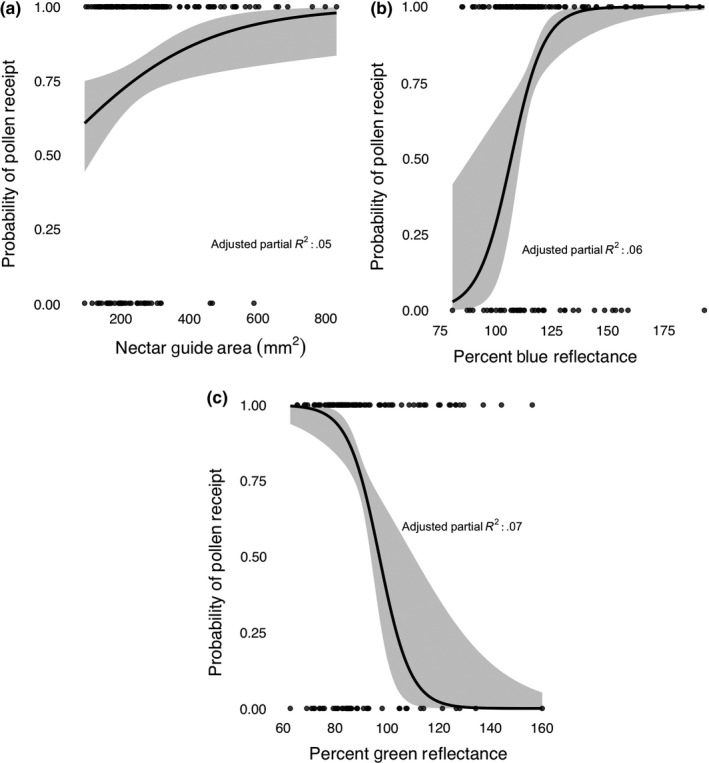
Predictor effect plots showing the effects of (a) nectar guide area (mm^2^) (b) blue mean petal reflectance, and (c) green mean petal reflectance on the probability of pollen receipt

To visualize the effects of nectar guide area and green/blue mean petal reflectance on pollen receipt we generated predictor effect plots (Figure 5), which provide graphical summaries for fitted regression models (Fox & Weisberg, 2018). The predictor effect displays in Figure 5 show the effects of nectar guide area, and blue and green mean petal reflectance on the probability of pollen receipt (receiving ≥1 pollen grain) holding all other variables equal (all the other continuous variables in the model are set to their mean value). Predictor effect plots are implemented in *R* in the “effects” package (Fox & Weisberg, 2018). We generated adjusted partial *R* ^2^ values using the “rsq.partial” function from the rsq package, and we report these in Figure 5.

### 3.3 Does the difference between petal color and background vegetation influence pollen receipt?

Flowers that appeared the most different from the background foliage in terms of green reflectance received the most pollen (*p* = .0001). Interestingly, the opposite pattern was observed for blue mean reflectance; flowers most similar to their background received significantly more pollen than flowers that were more distinct (*p* = .0003, Table 2b).

The combination of analyses conducted here indicates that flowers with high green reflectance tend to have low blue reflectance. If blue and green floral colors are developmentally correlated, then a pollinating insect could never encounter a flower with, for example, high blue and high green mean reflectance. Therefore, it may not be appropriate to try to disentangle their effects statistically.

## 4 DISCUSSION

This is among the first studies to examine sexual dimorphism between the functional sex stages of a bisexual flower (Davis et al., 2014; Jabbari et al., 2013). Here, in each of three functional sex stages of a common annual forb, we quantified a suite of floral traits that are visually distinguishable to pollinating insects and are known to influence pollinator attraction (in other species) (Glaettli & Barrett, 2008; Jones, 1996a; Leonard & Papaj, 2011; Rae & Vamosi, 2013). Our goals were to determine the effects of functional sex on petal size, color, and pattern in *C. unguiculata* and to establish which, if any, of these floral features influence pollinator visitation and effective pollen receipt in this widely studied species. We also synthesize our results to begin to understand how selection may generate sexual dimorphism in a sequential hermaphrodite such as *C. unguiculata*.

Significant differences between the functional sex stages of bisexual flowers may not represent the outcome of selection. However, two mechanisms have been proposed to promote the evolution of sexual dimorphism in flowering plants by natural selection: the avoidance of self‐fertilization (which may result in more attractive female‐phase flowers in protandrous species) and the maximization of sex‐specific fitness (which may result in more attractive male‐phase flowers) (Delph & Ashman, 2006). In this study, we found that functional sex is a significant source of variation in floral attraction traits in *C. unguiculata*, but our results do not align completely with either of these predictions.

In one of the only other studies to date to examine sexual dimorphism in a sequentially hermaphroditic plant species, Davis et al. (2014) found that female‐phase flowers of *Saponaria officinalis* (Caryophyllaceae) have larger petals and are “pinker” in color than male‐phase flowers. However, due to a change in floral presentation, female‐phase flowers have smaller corolla diameters than male‐phase flowers and attract significantly fewer pollinators. Pollinators of *S. officinalis* prefer whiter (less pink) flowers independent of the presence of stamens, nectar volume, and flower size (Davis et al., 2014). A strong pollinator preference for the floral phenotype associated with male sexual function in *S. officinalis* supports the hypothesis that sexual dimorphism may result in features that promote male fitness more than female fitness in a bisexual flower (at least in *S. officinalis*).

Our study revealed that sexual dimorphism is a significant source of variation in floral attraction traits in *C. unguiculata*. However, our separation of male function into two distinct stages and our examination of multiple components of flower color and pattern revealed a more complex division of attractive traits between the sexes.

### 4.1 Petal size and pattern

In *C. unguiculata,* the most substantial difference between male‐phase and female‐phase flowers was petal size. The petals of female‐phase flowers were 2‐3× larger than the male‐phase flowers from the same plant. Relatively large flowers have been associated with higher pollinator attraction and a subsequent increase in male and female reproductive success in other species (Galen, 2000; Glaettli & Barrett, 2008; Teixido, Barrio, & Valladares, 2016). However, nonsignificant and negative relationships between petal area and female fitness have also been reported (Barrio & Teixido, 2014; Delph & Ashman, 2006; Sáez et al., 2014; Teixido & Valladares, 2014; Wright & Meagher, 2004). In *C. unguiculata,* the sizes of petals and UV‐absorbing nectar guides were largest in female stage flowers (stage 3); however, petal area did not significantly affect pollen receipt.

A previous study (Mazer, Chellew, & Peach, 2019) revealed that field‐collected, senesced stigmas of *C. unguiculata* sampled from individuals with relatively large petals receive significantly more pollen than those sampled from small‐petaled individuals in some, but not all, populations. The stigmas sampled by Mazer et al. (2019) were harvested from fully intact individual plants after the flowers had senesced; receptive stigmas were therefore exposed to pollinators for more than 24 hr. By contrast, in the current study, individual flowers were removed from intact, greenhouse‐grown plants and then displayed to pollinators in the field, with stigmas harvested after two hours of exposure to pollinators. The difference between the Mazer et al. (2019) study and the field experiment described here with respect to the effect of petal area on pollen receipt may be due in part to this methodological difference. The amount of pollen received during the first two hours of a stigma's exposure to pollinators may not be strongly correlated with the total amount of pollen received by receptive stigmas. Alternatively, the difference between the two studies may reflect geographic variation in pollinator preference or the strength of pollinator‐mediated selection (Gómez, Perfectti, Bosch, & Camacho, 2009). In addition, petal area in *C. unguiculata* may be positively correlated with one or more other floral traits not measured by Mazer et al. (2019), but which may have promoted pollen receipt. Glaettli and Barrett (2008), Dudash Hassler, Stevens, & Fenster, (2011), and Eckhart (1991) similarly found that in *Sagittaria latifolia*, *Silene virginica*, and *Phacelia linearis*, respectively, flower/display size is positively correlated with pollinator visitation, but these studies did not examine the independent effects of additional components of floral color and pattern (that may be correlated with petal area) on pollen receipt or female reproduction.

In many wild species, relatively large flowers provide relatively large nectar rewards and are therefore considered “honest” signals to pollinators (Ashman & Stanton, 1991; Campbell, Waser, Price, Lynch, & Mitchell, 1991; Young & Stanton, 1990). In our study, we removed the nectar from flowers in order to detect the direct effects of other floral traits on pollen receipt. It is possible that pollinators visiting the experimental array preferentially approached large flowers from a distance, but close‐range floral cues (such as floral humidity or olfactory cues) indicated an absence of nectar (in which case petal size would not have been an honest signal of the available reward in this experiment). The removal of nectar may have therefore prevented us from detecting a pollinator preference for large flowers. The absence of nectar may have also influenced pollinator movement between flowers within the array, thus potentially further affecting pollen deposition.

The positive effect of UV nectar guide area on pollen receipt observed here corroborates previous studies that have reported a positive effect of UV floral guide presence, size, or proportion on pollinator visitation rate (Horth et al., 2014; Leonard & Papaj, 2011; Rae & Vamosi, 2013). UV bullseyes or UV nectar guides are often interpreted as guides that direct floral visitors toward a floral reward (Leonard & Papaj, 2011; Orban & Plowright, 2014). Recent research has shown that floral patterns can be seen by an insect and influence its behavior when it is in close proximity to a flower, often provoking an insect to land on and interact with the flower (Lunau, Unseld, & Wolter, 2009).

Another floral pattern that has been described as a pollinator attraction trait is the presence/size of petal spots (Glover, 2013; Jones, 1996a). A previous study of *Clarkia gracilis* found that pollinators in some locations had a slight preference for spotted (relative to unspotted) flowers (Jones, 1996a). However, in the current study, petal spot size did not have a significant effect on pollen receipt in *C. unguiculata,* so it was removed from the final model. It is often assumed that floral patterns help pollinators to discriminate among flowers and to identify the most rewarding ones (Hempel de Ibarra, Langridge, & Vorobyev, 2015). We do not know, however, whether petal spot size is correlated with nectar quantity or quality in this species. Moreover, the method we used to measure petal spot size did not allow us to determine the color of the petal spot. It is possible that a combination of petal spot size and color influences pollinator visitation in this species and we were unable to detect this preference.

Several studies have shown that floral patterns (such as petal spots and UV guides) may influence pollinator attraction (and pollen receipt). However, if we had only examined UV floral patterns we would have found that flowers with large UV‐absorbing nectar guides receive more pollen than those with small nectar guides, and female‐phase flowers have the largest nectar guides. However, because we examined visible flower color as well, we found that male‐phase flowers may be more attractive in terms of flower colors in the visible spectrum (Figure 4).

### 4.2 Petal color (blue, green, UV)

In *C. unguiculata*, flowers with relatively high mean blue reflectance or low mean green reflectance were more likely to receive pollen than flowers of alternative phenotypes. These features, however, do not increase linearly across functional sex stages (i.e., the expression of the “most attractive” phenotypes were not found exclusively in the female stage) (Figure 4).

Previous studies of bee color detection and preference have often used an achromatic background of green, gray, or white to detect pollinator color preferences (Bukovac et al., 2016; Giurfa, Núñez, Chittka, & Menzel, 1995; Hempel de Ibarra, Vorobyev, & Menzel, 2014; Shrestha, Dyer, Bhattarai, & Burd, 2014). However, Bukovac et al. (2017) determined that background colors may have a strong influence on which floral colors are detectable, and discriminable, for foraging bees. Specifically, for bees, the green photoreceptor may provide a high signal‐to‐noise ratio compared with the other photoreceptors as a means to distinguish flowers from green leaves (Chittka, Thomson, & Waser, 1999; Vasas, Hanley, Kevan, & Chittka, 2017). Our findings support this idea, as flowers that are more different from their background (in green reflectance) receive significantly more pollen than those with less distinct floral phenotypes (Table 2). While our study corroborates previous work that has shown that pollinators use green photoreceptors to distinguish flowers from leaves, the strong negative correlation between green and blue reflectance prevents us from determining whether there is truly an independent attractive function of blue reflectance or whether these color measurements are necessarily/developmentally correlated.

### 4.3 Pollen receipt as an estimate of pollinator visitation

Hove, Mazer, and Ivey (2016) found little evidence of pollen‐limited seed set in *C. unguiculata* within the Lake Isabella Region (Kern and Tulare counties, California, USA) from 2008 to 2010. Fruits produced by pollen‐supplemented and open‐pollinated flowers consistently exhibited levels of seed set. However, there was variation in pollen receipt and seed set within the open‐pollinated group, suggesting that some floral or inflorescence phenotypes were more attractive to pollinators than others (Hove et al., 2016).

While our goal was to understand the relationship between our focal floral traits and pollinator visitation, we measured pollen receipt as a proxy for the quantity and quality of pollinator visits. Observational measures of pollinator visitation often do not directly correlate with the deposition of pollen grains on stigmas (Alarcón, 2010; King, Ballantyne, & Willmer, 2013) because not all floral visitors are effective pollinators (Alarcón, 2010; Engel & Irwin, 2003; King et al., 2013). Consequently, estimating pollinator visitation using the number of pollen grains deposited on individual, receptive stigmas is a more realistic assessment of the pollination services received.

### 4.4 Support for more attractive female‐phase flowers

Female‐phase flowers produce the largest UV nectar guides, and nectar guide size is positively correlated with pollen receipt. This result supports the prediction that female‐phase flowers may evolve to be more attractive than male‐phase flowers to reduce the fitness costs associated with geitonogamous self‐fertilization. However, further pollination studies in the field, in multiple populations, would be required to determine the source of selection for sexual dimorphism in UV nectar guide size. To test this, we would compare rates of geitonogamous self‐fertilization among individuals (within each population) that exhibited natural or experimentally induced variation in UV nectar guide size. We could then ask the following: Does the amount of sexual dimorphism in UV nectar guide size (between male‐phase and female‐phase flowers on the same plant) affect the rate of self‐fertilization? In other words, do individuals with male‐phase and female‐phase flowers that look more different from each other experience higher rates of outcrossing (compared with individuals that exhibited a less pronounced sexual dimorphism)?

### 4.5 Support for more attractive male‐phase flowers

We did not find evidence for selection for more attractive male‐phase flowers in *C. unguiculata* (using the suite of visual floral traits examined here). Davis et al. (2014) found that pollinators preferred whiter flowers (compared with pink ones) and that male‐phase flowers were significantly whiter than female‐phase flowers. In this study, we also identified a pollinator preference for a specific color phenotype; however, it was a composite phenotype. Flowers with both high blue mean reflectance and low green mean reflectance received significantly more pollen than alternative color phenotypes. The flowers with the most attractive blue phenotype (highest blue mean reflectance) were stage 2 (male 2) flowers. However, the flowers with the most attractive green phenotype (low green mean reflectance) were flowers in stages 1 (male 1) and 3 (female). We found that blue mean petal reflectance and green mean petal reflectance explain nearly the same amount of variance in pollen receipt in the field (they have nearly identical adjusted partial *R* ^2^ values), so we cannot determine whether one color is more important for pollinator attraction than the other.

*C. unguiculata* is primarily pollinated by a group of specialist “*Clarkia* bees,” which have been observed collecting *Clarkia* pollen to consume as food. Male‐phase flowers of *C. unguiculata* may attract pollinators through a combination of signals, including colorful anthers and pollen. Given that we emasculated our experimental flowers and did not examine pollen export in this study, we could not evaluate this possibility.

While we did not find support for the prediction that male‐phase flowers may evolve to be more attractive than female‐phase flowers, we did find preliminary support for two ancillary predictions: (a) There were significant differences in petal area, blue, and green mean petal reflectance between the two male stages associated with the maturation of distinct and dimorphic sets of anthers in *C. unguiculata* (which supports our decision to consider them separately) and (b) the nonlinear pattern of flower color change across floral development (with peak reflectance in stage 2 flowers) suggests that flower color change is not just an inevitable byproduct of floral development in this species. If mean color reflectance values increased linearly from stages 1 to 3, then it would have been harder to rule out that flower color change was a selectively neutral byproduct of the expansion of petal cell vacuoles.

## 5 CONCLUSIONS

Overall, our results indicate that *C. unguiculata* flowers with green reflectance values that contrast most strongly with a typical leaf receive more effective visits from pollinators than flowers that are more “leaf‐like.” The size of the UV nectar guide also has an important attractive function in this species. Nevertheless, additional experimental field studies are required to determine whether the pollinators of *Clarkia* prefer the male‐phase (stage 1), the male‐phase (stage 2), or the female‐phase (stage 3) phenotype. Future work should also examine the effects of blue and green reflectance and UV pattern size on male fitness (pollen export) as well as on female fitness (pollen receipt and subsequent seed production and quality).

In this study, (a) we determined that functional sex (stage) is a source of variation in petal size, color, and pattern in *Clarkia unguiculata* (Onagraceae); (b) we found that blue and green mean petal reflectance, the size of the UV nectar guide, and the proportion of the petal occupied by the UV nectar guide significantly affect pollen receipt; and (c) our examination of multiple components of flower color and pattern revealed a more complex pattern of sexual dimorphism than we may have otherwise observed (had we examined fewer floral attraction traits). This study is among the first to examine sexual dimorphism between the functional sex stages of a bisexual flower.

Many wildflowers rely heavily on animal pollen vectors for successful reproduction, and decades of research have shown that pollinators respond to a variety of floral traits to locate and to access wildflowers and associated floral rewards (Chittka et al., 1999; Endress, 2011; Fenster, Armbruster, Wilson, Dudash, & Thomson, 2004). A pivotal discovery in explaining the diversity of flowering plants was that floral traits may evolve in response to specialized pollinators, which require particular floral traits in order to provide reliable pollination (Brosi, 2016; Fenster et al., 2004). Evolutionary theory predicts that, for this mutualism to be maintained, flowers should be morphologically constant (or very close to it) within populations and species, due to strong natural selection favoring genotypes that attract specific pollinators (Chittka et al., 1999; Waser, 1986). In spite of the prediction that wild species should harbor low levels of genetic variation in floral traits, many wild plant species (including *Clarkia unguiculata*, which relies primarily on pollinator specialists, the “*Clarkia* bees” [MacSwain et al., 1973]) exhibit tremendous intraspecific variation in petal size and floral color and pattern, in both the UV and visible portions of the spectrum. Extending our examination of sexual dimorphism to species characterized by dichogamous, bisexual flowers may improve our ability to explain the causes and consequences of intraspecific variation in wild and agricultural species.

## CONFLICT OF INTEREST

The authors have no competing interests to declare.

## AUTHORS' CONTRIBUTIONS

Contributions statement: Kristen Peach and Susan J. Mazer conceived the ideas and designed methodology; Kristen Peach, Jasen W. Liu, and Kristen Klitgaard collected the data and participated in analytical discussions; Kristen Peach and Jasen W. Liu analyzed the data; and Kristen Peach and Susan J. Mazer led the writing of the manuscript and the interpretation of the results. All authors contributed critically to the drafts and gave final approval for publication.

## Data Availability

The data that support the findings of this study are openly available in UC Santa Barbara Dash at https://doi.org/10.25349/D9QG65. Citation: Peach, Kristen; Mazer, Susan; Liu, Jasen; Klitgaard, Kristen (2019), Sexual dimorphism and sex‐specific floral attraction traits in a cosexual species, UC Santa Barbara Dash, Dataset, https://doi.org/10.25349/D9QG65

## Appendix 1

**Table A1:** Locations of wild populations of *Clarkia unguiculata* from which seeds were collected

Population name	Latitude	Longitude	Elevation (m)
Garrapata State Park	36.45499	121.91792	475
Bear Creek Rd.	37.16563	122.01796	651
Dark Canyon Rd	39.68256	121.39094	811
Auburn Recreation Area	38.93301	121.01162	364
Evey Canyon	34.16367	117.68258	704
Emerson Oaks Reserve	33.46826	117.07271	445
Matilija Creek	34.51474	119.38087	506
Wishon Drive	36.18794	118.67141	1,157

## Appendix 2

**Table A2:** Parameter estimates and standard errors for model results reported in Table 1 (main text) and a correlation matrix of focal traits

Term	Blue mean reflectance of the petal			Green mean reflectance of the petal			Ultraviolet mean reflectance of the petal		
	Estimate	SE	Prob> |t|	Estimate	SE	Prob> |t|	Estimate	SE	Prob> |t|
Population									
Auburn Road	−2.328	3.799	.541	−2.212	3.57	.536	−2.602	1.304	**.047**
Bear Creek	2.197	3.871	.571	3.573	3.637	.327	−2.03	1.394	.147
Dark Canyon	0.379	3.673	.917	1.673	3.452	.628	−1.092	1.258	.386
Evey Canyon	14.327	4.533	**.002**	11.603	4.26	**.007**	−0.112	1.432	.937
Emerson Oaks	−7.498	2.723	**.006**	−6.598	2.558	**.011**	−4.014	0.921	**<.0001**
Garrapata Park	−5.203	3.315	.112	−7.877	3.115	**.012**	1.917	1.161	.1
Matilija Creek	2.67	4.934	.589	2.186	4.637	.638	1.993	1.789	.267
Floral Stage 1	−4.009	1.792	**.026**	−0.87	1.684	.607	−1.579	0.721	**.029**
Floral Stage 2	6.533	1.787	**.0003**	5.692	1.679	**.001**	1.704	0.723	**.019**
Floral Sequence	−0.619	0.241	**.011**	−0.689	0.226	**.003**	−0.017	0.071	.806

Term	Petal area (mm^2^)			Nectar guide area (mm^2^)			Proportion nectar guide		
	Estimate	SE	Prob> |t|	Estimate	SE	Prob> |t|	Estimate	SE	Prob> |t|
Population									
Auburn Road	19.104	15.208	.211	32.429	8.829	**.0003**	0.049	0.013	**.0001** *
Bear Creek	−6.482	15.494	.676	34.102	9.462	**.0004**	0.052	0.013	**.0001** *
Dark Canyon	−33.186	14.704	**.025**	−4.794	8.539	.575	0.002	0.012	.868
Evey Canyon	−15.825	18.147	.384	−13.087	9.712	.179	−0.014	0.014	.31
Emerson Oaks	−21.852	10.899	**.046**	−19.973	6.239	**.0016**	−0.021	0.009	**.016** *
Garrapata Park	174.103	13.269	**<.0001**	5.285	7.874	.503	−0.028	0.011	**.011** *
Matilija Creek	−73.478	19.751	**.0003**	−19.959	12.142	.102	−0.015	0.017	.382
Floral Stage 1	−142.441	7.174	**<.0001**	−15.181	4.389	**.0007**	0.013	0.006	**.044** *
Floral Stage 2	26.879	7.156	**.0002**	2.158	4.416	.625	−0.004	0.006	.55
Floral Sequence	**−0.329**	**0.966**	.734	0.494	0.467	.291	0.002	0.001	**.023** *

* Indicates significant *P* values

## Appendix 3

Multispectral image generation and analysis—we converted a Panasonic LUMIX GX7 digital camera with LUMIX 14‐42mm II lens (http://www.panasonic.com, Kadoma, Osaka Prefecture, Japan) to a “full‐spectrum” camera using Life Pixel conversion services (http://www.lifepixel.com, Mukilteo, WA, USA). After conversion, we used two filters alternately to produce two images (which were later merged in ImageJ). The first image was created using a Baader U 2” UV‐pass filter (http://www.baader-planetarium.com, Mammendorf, Germany), which can be manually added to the camera to allow only ultraviolet (UV) light (300‐400nm with peak permeability at 350nm) to hit the sensor, thereby generating a UV image. To create the second image, we replaced the UV‐pass filter with a UV/infrared light blocking filter (http://www.uvroptics.com, UVR Defense Tech, USA), which allows only visible green, blue, and red light to pass through and hit the camera sensor. The human red channel is partitioned out of the final multispectral photograph such that pigment data from the red channel wavelengths are excluded from analysis.

Both photographs were taken at a station which included the full‐spectrum modified camera on a tripod with a locked camera position and a standardized light source (a single 70W Exo terro sunray™, http://www.exo-terra.com, Mansfield, MA, USA). The height and the angle of the light source do not change between photographs. The image frame included a fixed position ruler and Spectralon™ diffuse reflectance standard with a flat reflectance value of 20% across the spectrum (http://www.labsphere.com, North Sutton, NH, USA). The reflectance standard is used in the ImageJ software to control for unavoidable variation in the lighting environment throughout the study (Stevens et al., 2007). The Multispectral Image Analysis Toolbox plug‐in (Troscianko & Stevens, 2015) for ImageJ (Schneider et al., 2012) combines photographs taken in the visible and ultraviolet spectrum (into one multispectral image) and facilitates the extraction of objective measurements of color‐specific reflectance values and pattern. In all 32‐bit multispectral images, each pixel has a numeric value between 1 and 65,535, which represented an objective measurement of wavelength‐specific light reflectance. This value can be divided by 655.35 to attain a “percent reflectance.” We used this plug‐in to standardize and linearize the photographs and measure blue, green, and ultraviolet reflectance in different regions of the petal and determine the size of petal spots and nectar guides. A similar method of image acquisition and analysis was reported in detail earlier this year by Christian Verhoeven and colleagues (Verhoeven et al., 2018).

Pattern analysis (to determine petal spot size) was performed on each multispectral photograph using fast Fourier transform band‐pass filtering at different spatial scales from 2 pixels increasing exponentially with √2 to 600 pixels. This type of “granularity” pattern analysis has been used in a number of previous studies to analyze animal markings (Godfrey et al. 1987), cuttlefish camouflage (Barbosa et al., 2008; Chiao et al. 2009), and cuckoo eggshell pattern (Stoddard and Stevens 2010). For each “multispectral” image of a flower petal, we produced seven new images, each containing information at different spatial scales, by fast Fourier transforming the original image (Godfrey et al. 1987) and applying seven octave‐wide, isotropic band‐pass filters (Barbosa et al., 2008). These filters function like a sieve, capturing information at different spatial scales (different sized petal spots), with smaller filter sizes corresponding to larger (low spatial frequency) spots and larger filter sizes corresponding to smaller (high spatial frequency) spots. Adding together, the seven different filtered images produce a new image that is a close approximation to the original unfiltered image, with only a small loss of information. Analyzing these seven different images (“granularity bands”; Barbosa et al., 2008) allows us to quantify petal spot size and compare petal spot sizes between petals from different individuals. The granularity filtering approach is broadly based on well‐established principles of lower‐level vision and is supported by the neurophysiology of a range of vertebrate and invertebrate animal species (Campbell and Robson 1968; Godfrey et al. 1987; Stoddard and Stevens 2010).

**References for Appendix 3:**

Barbosa A, Mäthger LM, Buresch KC, Kelly J, Chubb C, Chiao CC,& Hanlon RT. (2008). Cuttlefish camouflage: the effects of substrate contrast and size in evoking uniform, mottle or disruptive body patterns. *Vision Research*, 48, 1242–1253.

Campbell FW, & Robson JG. (1968). Applications of Fourier analysis to the visibility of gratings. *Journal of Physiology*, 197, 551–556.

Chiao CC, Chubb C, Buresch KC, Siemann L, & Hanlon RT. (2009). The scaling effects of substrate texture on camouflage patterning in cuttlefish. *Vision Research*, 49, 1647–1656.

Godfrey D, Lythgoe JN, & Rumball DA. (1987). Zebra stripes and tiger stripes: the spatial frequency distribution of the pattern compared to that of the background is significant in display and crypsis. *Biological Journal of the Linnean Society*, 32, 427–433.

Schneider CA, Rasband WS, & Eliceiri KW. (2012). NIH Image to ImageJ: 25 years of image analysis. *Nature methods*, 9, 671–675.

Spottiswoode CN, & Stevens M. (2010). Visual modeling shows that avian host parents use multiple visual cues in rejecting parasitic eggs. *Proceeding of the National Academy of Sciences of the United States of America*, 107, 8672–8676.

Stevens M, Párraga AC, Cuthill IC, Partridge JC, & Troscianko J. (2007). Using digital photography to study animal coloration. *Biological Journal of the Linnean Society*, 90, 211–237.

Stoddard MC, & Stevens M. (2010). Pattern mimicry of host eggs by the common cuckoo, as seen through a bird's eye. *Proceedings of the Royal Society B*, 277, 1387–1393.

Troscianko J, & Stevens M. (2015). Image calibration and analysis toolbox – a free software suite for objectively measuring reflectance, colour and pattern. *Methods in Ecology and Evolution*, 6, 1320–1331.

Verhoeven C, Ren ZX, & Lunau K. (2018). False‐colour Photography: A Novel Digital Approach to Visualize the Bee View of Flowers. *Journal of Pollination Ecology*, 23, 102–118.

## Appendix 4

*Effects of Population on Floral Attraction Traits—*the ANOVAs revealed significant differences among population means with respect to blue (*F* = 2.70, *p* = .01), green (*F* = 2.80, *p* = .008), and UV petal reflectance (*F* = 5.84, *p* < .0001), petal area (F:26.81, *p* < .0001), and the area of the UV nectar guide (*F* = 5.73, *p* < .0001). The population x floral stage interaction has a significant effect on UV reflectance of the petal (*F* = 1.98, *p* = .02) (see Appendix 2 for parameter estimates).

*Effects of Floral Sequence on Floral Attraction Traits—*petals that were sampled from flowers at relatively distal positions on the primary stem exhibit significantly lower green and blue reflectance than those located at more basal positions. The floral sequence of the sampled flower has a significant and negative effect on blue (*F* = 6.57, *p* = .01) and green reflectance of the petal (*F* = 9.25, *p* = .002). There is no effect of floral sequence on UV reflectance of the whole petal, petal area, or nectar guide area. However, the floral sequence x population interaction does have a significant effect on blue (*F* = 2.38, *p* = .02) and green (*F* = 3.24, *p* = .003) mean reflectance of the petal and petal area (*F* = 4.95, *p* < .0001) (see Table S2 for parameter estimates).

**Table A4:** Summary of the ordinal logistic regression we conducted to determine the effects of floral stage, floral sequence, and population on petal spot size

Term	*df*	L‐R Chi‐square	Prob > ChiSq
Floral Sequence	1	0.9	0.34
Floral Stage	2	1.56	0.46
Population	7	22.2	**0.002** *
**Model**	10		**0.003** *

Term	Estimate	SE	Chi‐square	Prob > ChiSq
Petal Spot Size Bins				
2	−2.73	0.33	66.8	**<.0001** *
2.828	−1.30	0.24	29.42	**<.0001** *
8	−1.25	0.24	27.35	**<.0001** *
11.314	−1.22	0.24	26.34	**<.0001** *
16	−1.16	0.24	24.33	**<.00001** *
22.627	−1.09	0.23	21.43	**<.0001** *
32	−0.98	0.23	17.86	**<.0001** *
45.255	−0.72	0.23	10.26	**.0014** *
50	0.47	0.22	4.52	**.03** *
64	0.51	0.22	5.32	**.02** *
70.711	1.14	0.23	24.48	**<.0001** *
100	1.81	0.25	52.8	**<.0001** *
141.421	2.68	0.3	82.11	**<.0001** *
200	3.71	0.41	83.47	**<.0001** *
Floral Sequence	−0.02	0.02	0.93	.34
Floral Stage 1	0.18	0.17	1.11	.29
Floral Stage 2	0.01	0.17	0	.98
Population				
Auburn Road	−0.44	0.33	1.73	.19
Bear Creek	−0.54	0.36	2.23	.14
Dark Canyon	−0.59	0.32	3.26	.07
Emerson Oaks	−0.27	0.24	1.32	.25
Evey Canyon	0.33	0.37	0.82	.37
Garrapata State Park	0.37	0.3	1.55	.21
Matilija Creek	1.74	0.48	13.27	**.0003** *

* Indicates significant *P* values
